# Azemiopsin, a Selective Peptide Antagonist of Muscle Nicotinic Acetylcholine Receptor: Preclinical Evaluation as a Local Muscle Relaxant

**DOI:** 10.3390/toxins10010034

**Published:** 2018-01-07

**Authors:** Irina V. Shelukhina, Maxim N. Zhmak, Alexander V. Lobanov, Igor A. Ivanov, Alexandra I. Garifulina, Irina N. Kravchenko, Ekaterina A. Rasskazova, Margarita A. Salmova, Elena A. Tukhovskaya, Vladimir A. Rykov, Gulsara A. Slashcheva, Natalya S. Egorova, Inessa S. Muzyka, Victor I. Tsetlin, Yuri N. Utkin

**Affiliations:** 1 Shemyakin-Ovchinnikov Institute of Bioorganic Chemistry, Russian Academy of Sciences, ul. Miklukho-Maklaya 16/10, Moscow 117997, Russia; shelukhina.iv@yandex.ru (I.V.S.); mzhmak@gmail.com (M.N.Z.); chai.mail0@gmail.com (I.A.I.); garifulinaai@gmail.com (A.I.G.); natalyegorov@yandex.ru (N.S.E.); mis_kou@mail.ru (I.S.M.); vits@mx.ibch.ru (V.I.T.); 2 Branch of the Shemyakin-Ovchinnikov Institute of Bioorganic Chemistry, Russian Academy of Sciences, Pushchino 142290, Moscow Region, Russia; lobanov-av@yandex.ru (A.V.L); ikravchenko@bibch.ru (I.N.K.); katyarass@mail.ru (E.A.R.); mak401@gmail.com (M.A.S.); elentuk@mail.ru (E.A.T.); vladimirrykov@email.su (V.A.R.); slashcheva_ga@mail.ru (G.A.S.)

**Keywords:** nicotinic acetylcholine receptor, azemiopsin, preclinical studies, toxicity, pharmacokinetics, myorelaxant, Investigation of the preclinical profile of azemiopsin demonstrated its high affinity and specificity for muscle type nicotinic acetylcholine receptor as well as good muscle relaxant capacity. Toxicology studies in mice indicated that azemiopsin was well tolerated during chronic dosing and showed no immunotoxicity, allergenic or mutagenic activity, which made it a good candidate for application as a local muscle relaxant.

## Abstract

Azemiopsin (Az), a linear peptide from the *Azemiops feae* viper venom, contains no disulfide bonds, is a high-affinity and selective inhibitor of nicotinic acetylcholine receptor (nAChR) of muscle type and may be considered as potentially applicable nondepolarizing muscle relaxant. In this study, we investigated its preclinical profile in regard to in vitro and in vivo efficacy, acute and chronic toxicity, pharmacokinetics, allergenic capacity, immunotoxicity and mutagenic potency. The peptide effectively inhibited (IC_50_ ~ 19 nM) calcium response of muscle nAChR evoked by 30 μM (EC_100_) acetylcholine but was less potent (IC_50_ ~ 3 μM) at α7 nAChR activated by 10 μM (EC_50_) acetylcholine and had a low affinity to α4β2 and α3-containing nAChR, as well as to GABA_A_ or 5HT_3_ receptors. Its muscle relaxant effect was demonstrated at intramuscular injection to mice at doses of 30–300 µg/kg, 30 µg/kg being the initial effective dose and 90 µg/kg—the average effective dose. The maximal muscle relaxant effect of Az was achieved in 10 min after the administration and elimination half-life of Az in mice was calculated as 20–40 min. The longest period of Az action observed at a dose of 300 µg/kg was 55 min. The highest acute toxicity (LD_50_ 510 μg/kg) was observed at intravenous injection of Az, at intramuscular or intraperitoneal administration it was less toxic. The peptide showed practically no immunotoxic, allergenic or mutagenic capacity. Overall, the results demonstrate that Az has good drug-like properties for the application as local muscle relaxant and in its parameters, is not inferior to the relaxants currently used. However, some Az modification might be effective to extend its narrow therapeutic window, a typical characteristic and a weak point of all nondepolarizing myorelaxants.

## 1. Introduction

A linear peptide azemiopsin (Az) isolated from the *Azemiops feae* viper venom contains no disulfide bonds [1] and can be easily prepared by peptide synthesis. It is a high-affinity and selective inhibitor of muscle-type nicotinic acetylcholine receptor (nAChR) involved in fast synaptic signal transduction at nerve-muscle junction [2]. These receptors are well-known targets for muscle relaxant drugs (e.g., [3]). Muscle relaxants reduce the tone of the skeletal muscle with a decrease in motor activity up to complete immobilization. These drugs are generally classified into central muscle relaxants, which disrupt the transmission of excitation in the central nervous system and muscle relaxants of peripheral action that primarily and specifically disturb neuromuscular transmission.

Peripheral muscle relaxants can affect the signal transmission both at the presynaptic and postsynaptic membrane of the neuromuscular junction. The drugs acting at postsynaptic membrane are classified into depolarizing and nondepolarizing muscle relaxants (NMR). The action of depolarizing muscle relaxants (e.g., succinylcholine) is based on the persistent depolarization of the postsynaptic membrane, which makes impossible the propagation of the action potential and causes relaxation of the muscle fiber. NMRs (such as *d*-tubocurarine, atracurium, rocuronium) block the binding of acetylcholine to nAChR and disturb its function [4].

Nowadays, the muscle relaxants are used generally during large operations in order to achieve relaxation of the muscles (especially the abdomen) and thereby facilitate surgical manipulation. For most operations, the basic condition is a good relaxation of the striated muscles. NMRs are usually administered during anesthesia to facilitate endotracheal intubation and/or to improve surgical conditions. Currently, clinical practice cannot do without them. Muscle relaxants allowed reducing the depth of anesthesia and better controlling the conditions of the body’s systems. In addition to anesthesiology, muscle relaxants have found application in traumatology and orthopedics for muscle relaxation in the treatments of dislocations, fractures, diseases of the back and ligament. Short-acting drugs in combination with general anesthetic agents are often used to facilitate laryngoscopy, bronchoscopy and esophagoscopy. NMRs are applied parenterally, almost always injected intravenously.

It should be noted that NMRs have undesirable side effects, primarily associated with their effects on the autonomic nervous system and with the release of histamine. The reason for these effects is the insufficient selectivity of low molecular NMR for muscle-type nAChR. Moreover, a number of side effects are due to blockade or activation of the muscarinic acetylcholine receptor [5,6]. Because of these side effects, *d*-tubocurarine is practically not applied today.

To treat conditions where muscles spasms and spasticity are a problem, muscle relaxants are also used. Muscle spasms are caused by an involuntary contraction of the muscles, which is often painful and causes difficulty in performing everyday tasks. Spasticity occurs when a muscle contracts and remains in this tight position, becoming very stiff and almost impossible to use. In cases like this, muscle relaxants are used to control stiffness and involuntary movements. They are used to treat so-called muscle dystonia. Dystonia is defined by Dystonia Medical Research Foundation as “a movement disorder characterized by sustained or intermittent muscle contractions causing abnormal, often repetitive, movements, postures, or both. Dystonic movements are typically patterned, twisting and may be tremulous. Dystonia is often initiated or worsened by voluntary action and associated with overflow muscle activation” [7]. Examples of muscle dystonias are blepharospasm (involuntary squinting), cervical dystonia (torticollis), spasticity (hypertonicity) of skeletal muscle, writer’s cramp, foot dystonia, etc. The problem of constant high tone of particular groups of muscles also exists at spastic form of cerebral palsy. Currently, according to the European (http://dystonia-europe.org) and American (http://www.dystonia-foundation.org/) dystonia societies, the number of patients with various forms of dystonia is 500,000 in Europe and 300,000 in the United States. According to the Research Foundation for Cerebral Palsy Associations (UCPA: http://www.ucp.org/), there are approximately 760,000 patients with this disease in the United States. In Russia, the number of patients with muscular dystonia is estimated at 80,000–140,000 people and cerebral palsy—150,000–200,000.

Peripheral muscle relaxants have become medications for the treatment of muscle dystonia. Historically, the first applied in clinical practice peripheral relaxants were low-molecular alkaloids. After these first low-molecular cholinergic blockers, showing a number of side effects but being used up to the present time, the botulinum toxin has appeared. Currently, the main method of treatment for muscular dystonia and spastic form of cerebral palsy is the injection of botulinum toxin into the muscles involved in hyperkinesis. In 1989, the “BOTOX” (one of the drugs based on botulinum toxin) has been approved by the FDA for the treatment of blepharospasm, in 2000—cervical dystonia, in 2010—spasticity at the elbow, wrist and fingers. The clinical effect is achieved in 85–90% of cases and lasts 2–3 months, however patients are in need of repeated administration of the drug: for spastic torticollis—2 injections per year, blepharospasm—3–4, cerebral palsy—2 times a year. With the apparent effectiveness of “BOTOX”, there are a number of disadvantages associated with side effects, which include itching, burning, swelling at the injection site, in some patients there is a general muscle weakness during the first two weeks after application of the preparation, antibody formation shows in 3–10% of patients. Even the duration of its effect is a disadvantage, since it does not allow promptly adjusting the dose of the drug in accordance with individual tolerability. A significant drawback of the drug is associated with the mechanism of action of botulinum toxin at the molecular level.

Negative moments of this mechanism of action (and especially its duration) are progressive atrophy of muscle fiber with a decrease in the average diameter of the fiber, scattering of nAChRs from the site of the synapse and a decrease in the activity of synaptic acetylcholinesterase. On rabbits, it was shown that after the injections of botulinum toxin that lasts for six months, the reduction in muscle mass could reach 76% and the contractile fibers could be replaced by fatty tissue elements. Currently, there are no medicines capable of replacing BOTOX in the treatment of muscular dystonia in the world. Therefore, the task of creating new effective drugs that do not have side effects, for local therapy of muscular dystonia is extremely urgent.

Muscle relaxants of peptide nature may be considered as alternatives to low-molecular alkaloids with a large number of side effects and to extremely toxic protein botulinum toxin. Peptides are not xenobiotics and, as a rule, have high selectivity to specific targets, which is due to the very nature of peptide-protein recognition. The natural source of such peptides has always been the animal venoms, especially the venoms of molluscs and snakes [8]. In the venoms of molluscs and snakes, polypeptide and peptide compounds acting on neuromuscular transmission have been identified and potentially can be regarded as agents for the treatment of muscular dystonia. In particular, the discovery and characterization of Az, blocking the neuromuscular transmission, open the possibility for the development of a novel muscle relaxant. As an inhibitor of muscle nAChR, Az may be regarded as a potentially applicable muscle relaxant itself [9]. This paper reports the results of preclinical studies, including single- and repeated-dose toxicities, immunotoxicity, pharmacokinetic and other studies. Overall, the results obtained demonstrate that Az has good drug-like properties.

## 2. Results

### 2.1. Az Synthesis

**Figure 1:**
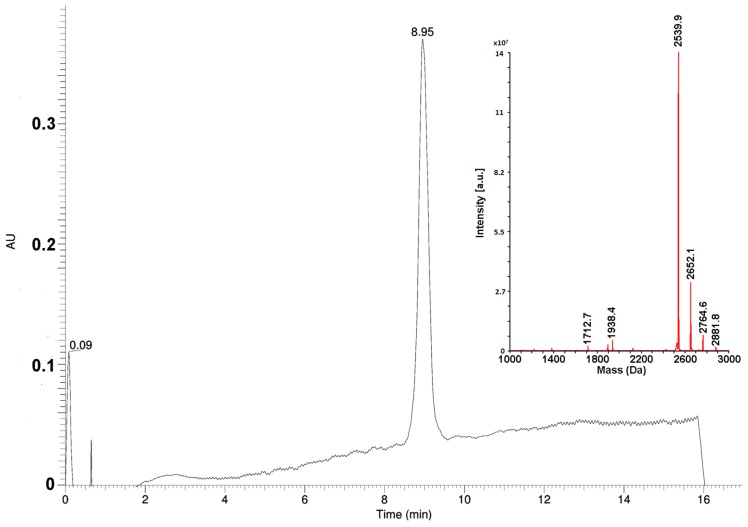
Analytical UPLC-MS on Phenomenex Aeris PEPTIDE XB-C18 column (1.7 µm, 2.1 × 150 mm) using a linear acetonitrile gradient from 10 to 35%. Inset. Deconvoluted mass-spectrum of Az sample obtained after the final purification step. a.u., arbitrary unit.

The Az peptide was prepared by a solid phase synthesis using a general Fmoc-strategy. The principal scheme was adopted from [10]. However, to minimize the consumption of materials for large-scale synthesis needs, all steps in the procedure were optimized and standardized. Thus, to determine the optimal excesses of amino acid derivatives, pilot experiments were performed. It was found, that for the first stage of the coupling of the C-terminal proline residue to a polymer, the 5-fold excess of the protected amino acid was necessary. For the coupling of the subsequent residues, the 4-fold excesses were enough. The couplings of the seventh and subsequent residues required the increase of reaction time from 1 to 2 h. The modified scheme of synthesis allowed to avoid unnecessary reagent consumption, in particular, of expensive protected amino acids. Optimization experiments showed that the repeated condensations were necessary only for coupling of residues His9 and Pro15. The amounts of solvents and coupling reagents were optimized as well, reducing their consumption by several times. Large-scale purification of the peptide was performed in two steps. Ion exchange chromatography on a weak cation exchanger under moderately basic conditions was used at the first step. It allowed to obtain the peptide with purity of about 90% without any organic solvent consumption. To meet pharmacopeia specifications, the final purification step was carried out by a reversed-phase HPLC, increasing the substance purity to greater than 97% (Figure 1). Using the optimized procedure, the final product, Az, was obtained with 20% yield.

### 2.2. Efficacy and Specificity of Az In Vitro

**Figure 2:**
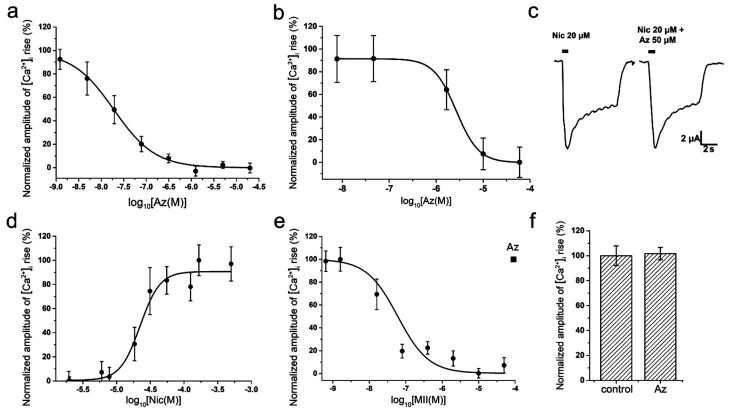
Interaction of Az with muscle and neuronal nAChRs. Inhibitory curves of Az action on ACh (30 and 10 μM)-evoked intracellular calcium concentration ([Ca^2+^]_i_) rises in neuroblastoma Neuro2a cells expressing (**a**) muscle α1β1εδ and (**b**) α7 nAChRs, respectively. (**c**) Representative nicotine (Nic)-induced current traces through α4β2 nAChR and (**d**) dose-response curve of [Ca^2+^]_i_ amplitude rise in neuroblastoma SH-SY5Y cells expressing α3-containing nAChRs in response to different concentrations of Nic. There are no inhibitory effects of Az on Nic-evoked (**c**) ion currents and (**f**) calcium responses mediated by α4β2 and α3-containing nAChRs, respectively (*p* > 0.05, Mann–Whitney U test). (**e**) Inhibitory curve of α-conotoxin MII action on Nic (100 μM)-evoked [Ca^2+^]_i_ rise in SH-SY5Y cells expressing α3-containing nAChRs. Each point represents data obtained from 4 independent experiments (mean ± SEM).

To study a specific activity and selectivity of Az in vitro, two different methods were used: electrophysiological method of two-electrode voltage-clamp on *Xenopus* oocytes and calcium imaging using the genetically encoded calcium sensor Case12 or the low-molecular weight calcium indicator Fluo-4. In calcium imaging experiments on mouse muscle type α1β1εδ nAChR, Az showed a high inhibitory activity in nanomolar range (IC_50_ = 19 ± 8 nM, Figure 2a). Az also manifested ability to interact with the human neuronal homopentameric α7 nAChR but with a much lower affinity (IC_50_ = 2.67 ± 0.02 μM, Figure 2b). It should be mentioned, that the corresponding cellular calcium responses were provoked by acetylcholine (ACh) at concentrations of 30 μM (EC_100_ on muscle nAChR) and 10 μM (EC_50_ on α7 nAChR), respectively [11]. Electrophysiology experiments discovered no influence of Az on ion currents induced by 20 μM nicotine in rat neuronal heteromeric α4β2 nAChR at a concentration up to 50 μM (Figure 2c). Besides, no Az activity against human neuronal heteromeric α3-containing nAChRs (α3β2, α3β4, etc.) expressed in neuroblastoma SH-SY5Y cells was detected by calcium imaging at a concentration up to 100 μM (Figure 2f). In control experiments using the same cellular system, calcium responses induced by 100 µM nicotine (EC_50_ = 22 ± 2 µM, Figure 2d) were successfully inhibited by α-conotoxin MII (IC_50_ = 60 ± 4 nM, Figure 2e), a specific antagonist of α3-containing nAChRs. In sum, these data demonstrated high selectivity of the Az action on muscle nAChR. 

**Figure 3:**
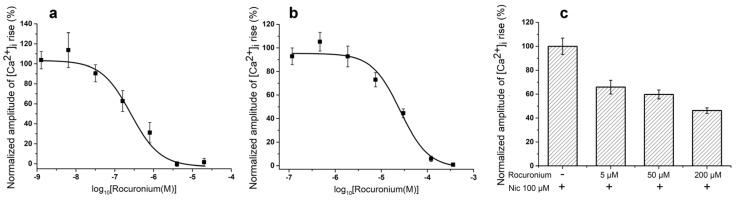
Interaction of rocuronium with muscle and neuronal nAChRs. Inhibitory curves of rocuronium action on ACh (30 and 10 μM)-evoked intracellular calcium concentration ([Ca^2+^]_i_) rises in neuroblastoma Neuro2a cells expressing (**a**) muscle α1β1εδ and (**b**) α7 nAChRs, respectively. Inhibition of [Ca^2+^]_i_ amplitude rise induced by Nic (100 μM) in neuroblastoma SH-SY5Y cells expressing α3-containing nAChRs by different concentrations of rocuronium (**c**). Each point represents data obtained from 4 independent experiments (mean ± SEM).

For comparison, we have tested NMR rocuronium in some in vitro experiments and found that at muscle type receptor it was less effective than Az, IC_50_s being 257.06 ± 95.54 nM and 19 ± 8 nM for rocuronium and Az, respectively (Figure 2a and Figure 3a). At the human neuronal homopentameric α7 nAChR rocuronium showed also lower affinity with IC_50_ of 25.69 ± 4.5 μM (Figure 3b) as compared to 2.67 ± 0.02 μM for Az (Figure 2b). In contrast to Az, at concentration up to 100 μM (Figure 2f) manifesting no activity against human neuronal heteromeric α3-containing nAChRs (α3β2, α3β4, etc.) expressed in neuroblastoma SH-SY5Y cells, rocuronium dose-dependently inhibited these nAChR subtypes (Figure 3c). At 200 μM rocuronium inhibited Nic induced currents by about 60% (Figure 3c).

### 2.3. In Vivo Efficacy Tests

#### 2.3.1. In Vivo Az Efficacy

**Figure 4:**
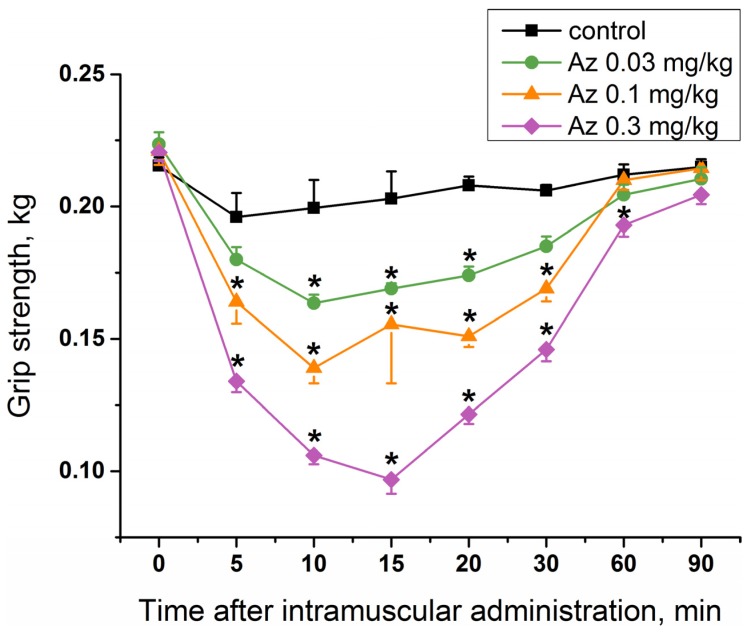
Muscle relaxant effect of Az. The time courses of a grip strength of mouse (ICR males) forelimbs at 0–90 min after Az (0.03, 0.1 and 0.3 mg/kg) or normal saline (control) intramuscular administration. The results are presented as mean values ± SEM, *n* = 10. Significant differences in the forelimb strength were revealed between control and experimental groups (one-way repeated measures ANOVA, * *p* < 0.05).

To study a specific activity of Az as an agent blocking neuromuscular transmission for the treatment of muscular dystonia, its effect on mouse muscular strength was estimated. Single administration of Az in the muscles of the forelimbs at doses of 0.03, 0.1 and 0.3 mg/kg caused a significant decrease in their muscular strength (Figure 4), while the dose of 0.01 mg/kg was not effective (Figure S1). The longest period of Az action was observed for a dose of 0.3 mg/kg and was maintained for 55 min from the 5th to the 60th minute after its administration. The maximal muscle relaxant effect of Az for all the doses studied was achieved 10 min after its administration and was preserved until the 30th minute. The dose of 0.03 mg/kg was considered as an initial effective dose. The average effective dose at the maximum response point (10 min after injection) was 0.09 mg/kg. These in vivo data showed the good muscle relaxing properties of Az.

#### 2.3.2. In Vivo Rocuronium Efficacy

**Figure 5:**
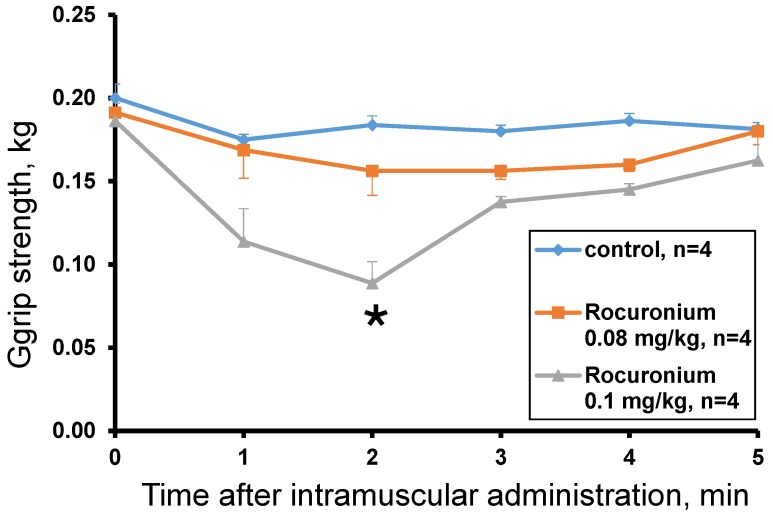
Muscle relaxant effect of rocuronium. The time courses of a grip strength of mouse (ICR males) forelimbs at 0–5 min after rocuronium (0.08, 0.1 mg/kg) or normal saline (control) intramuscular administration. The results are presented as mean values ± SEM, *n* = 4. Significant differences in the forelimb strength were revealed between control and experimental groups (one-way repeated measures ANOVA, * *p* < 0.05).

The rocuronium effect was studied at doses of 0.13 mg/kg, 0.1 mg/kg and 0.08 mg/kg. The dose of 0.13 mg/kg was lethal and after 60 s the animal lost muscle tone (grip strength = 0 kg) and the ability to move. The introduction of rocuronium at doses of 0.08 and 0.1 mg/kg did not cause the death of animals and showed a dose-dependent decrease in muscle tone within the first 5 min after administration. A statistically significant effect compared to the control was observed 2 min after administration of rocuronium at a dose of 0.1 mg/kg (Figure 5). At the 3rd minute after the administration, muscle strength began to recover and did not significantly differ from the control. At a dose of 0.08 mg/kg no statistically significant difference from control was found and only a tendency to decrease the muscular strength was observed with the greatest effect at the 2nd and 3rd minutes (Figure 5).

### 2.4. Pharmacokinetics of Az

To study a pharmacokinetics of Az, its radioiodinated ^125^I-labeled analog ([^125^I]-Az) was prepared.

#### 2.4.1. Preparation of [^125^I]-Az

In position 9, Az molecule contains a histidine residue which may be subjected to electrophilic iodination as published earlier [12]. However, two tryptophan residues (Trp3 and Trp4) might be oxidized under iodination conditions complicating the isolation of the target iodinated product. To overcome this problem, both tryptophan residues were protected by formylation. Diformyl-Az was iodinated following a standard chloramine protocol, optimized to obtain a better yield of iodinated peptide [13]. For the iodination reaction, a preliminary screening of the reaction conditions at which the pH and the substrate/chloramine ratio varied was carried out. It was not possible to obtain radioiodinated Az derivative containing only one iodine atom, therefore di-iodinated analogue was prepared. The complete iodination was achieved at pH 6.8 with 3 equivalents of iodide and 2.2 equivalents of chloramine T relative to peptide. The di-iodinated product was purified by HPLC and deprotected under alkaline conditions, followed by alkali neutralization. Analytical HPLC showed full removal of protecting groups, so the product was used further without additional purification.

#### 2.4.2. Pharmacokinetics Studies

Single intravenous (iv) and intramuscular (im) administrations of [^125^I]-Az at doses of 0.25 and 0.50 mg/kg to male ICR mice were performed. No lethality was observed after [^125^I]-Az injections at these doses. The main pharmacokinetic parameters such as the area under a pharmacokinetic curve (AUC(0 → t)), the maximum Az concentration in mouse blood (C_max_) and its excretion half-life T_1/2_ were determined (Table 1), allowing to evaluate the processes of excretion and elimination of the peptide.

**Figure 6:**
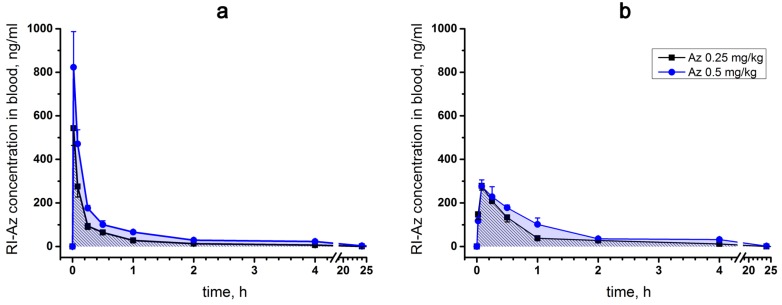
Pharmacokinetic curves of [^125^I]-Az concentration in blood of male ICR mice 0–24 h after its (**a**) intravenous and (**b**) intramuscular administration at doses of 0.25 and 0.5 mg/kg. The results are presented as mean values ± SD, *n* = 5 (each plot point represents mean results for five animals).

For a single intravenous injection, the maximum concentration (C_max_) of [^125^I]-Az in mouse blood was observed 1 min after injection and its excretion half-life (T_1/2_) was estimated as 15–20 min (Figure 6a). With intramuscular administration, the maximum [^125^I]-Az concentration (C_max_) was achieved within five minutes and the parameter T_1/2_ was calculated as 20–40 min (Figure 6b). In both modes of administration, the drug was almost completely removed from the free blood flow during 24 h (Figure 6a,b). A greater maximum drug concentration (C_max_) was observed at an intravenous route of administration than at intramuscular injection.

### 2.5. Acute Toxicity Tests

#### 2.5.1. Acute Toxicity of Az

The acute toxicity of Az after intraperitoneal administration to mice was determined earlier [1], the LD_50_ value was 2.57 ± 0.27 mg/kg. In the present work, we studied Az acute toxicity to male ICR mice after intravenous and intramuscular administration. The LD_50_ value was estimated as 0.51 ± 0.06 mg/kg after Az intravenous administration.

A single intramuscular Az injection at doses of 0.8, 0.775, 0.75 and 0.725 mg/kg resulted in a dose-dependent death of the mice. Death in 100% of cases was detected at a dose of 0.8 mg/kg. The doses of 0.775 and 0.75 mg/kg resulted in the death of 4 animals out of 5. At a dose of 0.725 mg/kg, 3 animals died out of 5 and at 0.7 mg/kg all mice were alive. The death of animals at doses of 0.725, 0.75, 0.775, 0.8 mg/kg was observed 19.7 ± 5.9, 28.8 ± 16.0, 15.8 ± 4.3 and 12.2 ± 1.8 min after Az administration, respectively. Based on these data, LD50 of 0.732 ± 0.13 mg/kg was calculated for Az at intramuscular injection. The maximal tolerant dose was 0.7 mg/kg after its intramuscular injection. No toxicity signs were observed at a dose of 0.3 mg/kg and lower used for grip strength tests.

Intramuscular injection of Az was accompanied by external signs of intoxication, the severity of which was dose-dependent. Visible toxic signs appeared 5–7 min after administration and were characterized by impaired coordination of movements and loss of muscle tone, decreased motor activity, impaired breathing, decreased response to external stimuli. Maximal manifestations of intoxication were noted between 10 and 20 min after administration and were characterized by loss of motor activity, a lacunar posture or posture on the side, loss of muscle tone, loss of response to external stimulation, delayed or intermittent breathing, coma. Mice almost completely recovered within 60 min after injection. After this period, the animals showed a decrease in motor activity and muscle tone. Complete recovery from the toxic effect of large doses occurred 24 h after administration. No function disturbances caused by Az large doses were observed 14 days after the administration.

At intramuscular administration way, a maximal tolerated dose (MTD) was determined. MTD is the highest dose of a drug that does not cause unacceptable side effects. In mice, MTD for Az was determined as 0.7 mg/kg; no lethality was observed at this dose. The doses up to 0.5 mg/mL used for the in vivo tests were lower than MTD of 0.7 mg/kg and induced no lethality as well.

#### 2.5.2. Acute Toxicity of Rocuronium

Injection of rocuronium at a dose of 0.13 mg/kg caused the death of the test animal. The first signs of intoxication were detected 30 s after injection. The impaired coordination of movements, decreased muscle tone, decreased motor activity, increased respiratory movements, convulsions were observed. The severity of toxic disorders rapidly increased. After 60 s, the animal lost muscle tone (grip strength = 0 kg) and the ability to move, the frequency of respiratory movements decreased, the animal fell into a coma and then died 11.5 min after injection. Rocuronium at doses of 0.08 and 0.1 mg/kg did not cause the death of animals. A dose of 0.08 mg/kg did not induce visible signs of toxicity, while 0.1 mg/kg caused discoordination of movements, decreased muscle tone, gait disturbance, decrease or short-term loss of motor activity, increased respiratory movements, vocalization (in one animal). These toxicity signs disappeared in 3–4 min after drug administration.

### 2.6. Subchronic Toxicity of Az

For the study of the subchronic toxicity, the number of animals was increased in comparison with acute toxicity tests, because many biochemical, histological and other parameters needed to be measured. In order to reveal a statistically significant difference in these parameters, large groups of animals were used. For the studies, larger animals (rats) were used for experimenting with long-term administration of the drug and studying many blood parameters, this might be difficult for small animals such as mice.

On the basis of acute toxicity results a repeated dose 14-day intramuscular toxicity study was performed. During the 14-day period of Az administration at doses of 0.1 and 0.5 mg/kg, it did not cause any evident signs of toxicity in male or female Sprague Dawley rats. The increase in the mean body weight of animals and in the food intake did not differ significantly between the experimental and control groups.

A 14-day Az treatment at a dose of 0.1 mg/kg did not lead to any changes in the hemogram of experimental animals relative to control ones. However, in male rats at a dose of 0.5 mg/kg Az caused a statistically significant increase (*n* = 6, *p* < 0.05, Kruskall-Wallis ANOVA on ranks) in the number of platelets (810 ± 39 g/L) relative to the control level (729 ± 22 g/L). Two weeks after the 0.5 mg/kg Az administration the level of platelets was still slightly increased but non-significantly. There were no differences in the hemograms of female rats receiving Az at both doses and the control group.

A few biochemical parameters of rat blood serum were changed at the end of 28-day subchronic toxicity experiment (14 days of Az administration and 14 days of its withdrawal) in comparison to the control animal group. In the blood serum of males receiving Az at a dose of 0.5 mg/kg, a significant decrease in the mean level of triglycerides (0.97 mmol/L vs. 1.25 mmol/L in control, *n* = 6, *p* < 0.05, Kruskall-Wallis ANOVA on ranks) was observed. In a group of female rats treated with 0.1 mg/kg of Az, the levels of cholesterol (2.82 ± 0.36 mmol/L vs. 3.55 ± 0.39 mmol/L in control) and calcium (3.09 ± 0.06 vs. 3.25 ± 0.09 mmol/L in control) were reduced significantly (*n* = 6, *p* < 0.05, Kruskall-Wallis ANOVA on ranks).

In two weeks after the Az administration the rats were euthanized. Post-mortem necropsy of animals did not reveal any abnormalities in the anatomy or in the absolute weight of their internal organs. However, some differences in a relative heart weight of male rats were observed. Thus, the mean relative heart weight in a rat group treated with Az at a dose of 0.5 mg/kg was significantly (*n* = 6, *p* < 0.05, Kruskall-Wallis ANOVA on ranks) reduced (0.352 ± 0.033%) in comparison with the control group of animals (0.428 ± 0.086%). Histological analysis was performed for the following organs and tissues: liver, stomach, kidneys, adrenal glands, lungs, heart, spleen, thymus, submandibular lymph nodes, ovaries, testes, brain, femoral muscle of the right and left paws (the injection site). No pathological changes were observed in the organs examined.

All observed changes in biochemical, hematological and other tested parameters were among physiologically normal variations for Sprague Dawley rats and did not indicate Az toxicity [14]. Thus, during 14-day intramuscular administration of Az in two doses of 0.1 and 0.5 mg/kg, which are similar to the expected therapeutic doses, to female and male rats with a two-week withdrawal period the peptide showed no significant toxicity.

### 2.7. Immunotoxicity of Az

To study the possible immunotoxicity of Az, its effects on

(1) cellular immunity (the delayed-type hypersensitivity reaction),
(2) immune response to a standard antigen and
(3) phagocytic activity of peritoneal macrophages was evaluated.

All three tests were carried out after 7-day intramuscular Az administration at doses of 0.15 and 0.5 mg/kg to male ICR mice. Control animals were treated with the sodium chloride physiological solution (normal saline).

**Figure 7:**
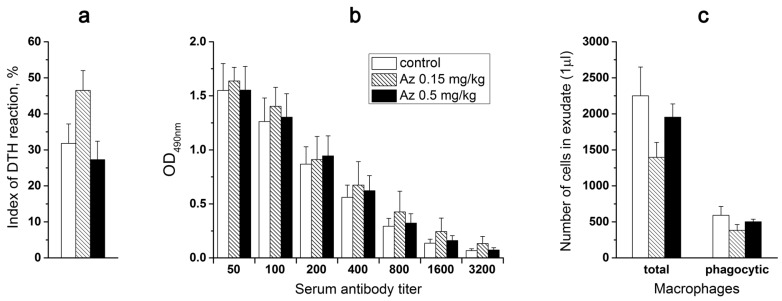
The influence of Az on immune system. The effect of 7-day intramuscular Az (0.15 and 0.5 mg/kg) administration to male ICR mice (**a**) on their delayed-type hypersensitivity (DTH) to a specific antigen (10 mM trinitrobenzenesulfonic acid) manifested by paw edema, (**b**) on their immune response to a bovine serum albumin (BSA) and (**c**) on phagocytic activity of their peritoneal macrophages. (**a**) The presented indexes of DTH reactions reflect the normalized difference in the weights of treated and control mouse hind paws. (**b**) The titers of IgG in blood serum of mice from experimental and control groups after their standard immunization with BSA are presented. (**c**) The number of total and phagocytic (engulfing ink particles) macrophages in 1 μL of peritoneal exudate isolated from experimental and control animals. In all three tests, there was no significant difference between control and experimental animals (*p* > 0.05, Kruskall-Wallis ANOVA on ranks) in the parameters studied. The results are presented as mean values ± SEM, *n* = 5–10.

To probe the Az influence on cellular immunity, the mice were primarily immunized subcutaneously (sbc) at the base of a tail with trinitrobenzenesulfonic acid (10 mM, 200 μL) and secondarily after 6 days with the same agent (50 μL) in the left hind paw. Simultaneously, the physiological solution (50 μL) was injected into the right control hind paw. Next day after the second immunization, the weight of left and right hind paws were compared and edema of the experimental paws was revealed in all animal groups (Figure 7a). However, Az treatment did not cause any significant changes in the degree of the observed edema, showing no influence on cellular immunity in mice (Figure 7a).

In the next test, the effect of a 7-day Az administration on mouse immune response to a bovine serum albumin (BSA) was determined. The mice from one control and two experimental groups were routinely immunized with BSA in two steps (1st and 10th days) and after a week the corresponding IgG titers were evaluated in mouse blood serum (Figure 7b). The obtained results did not demonstrate any significant difference in the immune response of mice treated with Az (0.15 and 0.5 mg/kg) or with the physiological solution (Figure 7b).

Az at doses of 0.15 and 0.5 mg/kg also did not significantly change the phagocytic activity (engulfing ink particles) of peritoneal macrophages, which were isolated from experimental animals, vs. control ones (Figure 7c). Thus, in all three tests no significant changes in the studied parameters of the immune system in animals receiving Az during a week were observed.

### 2.8. Allergenicity of Az

**Figure 8:**
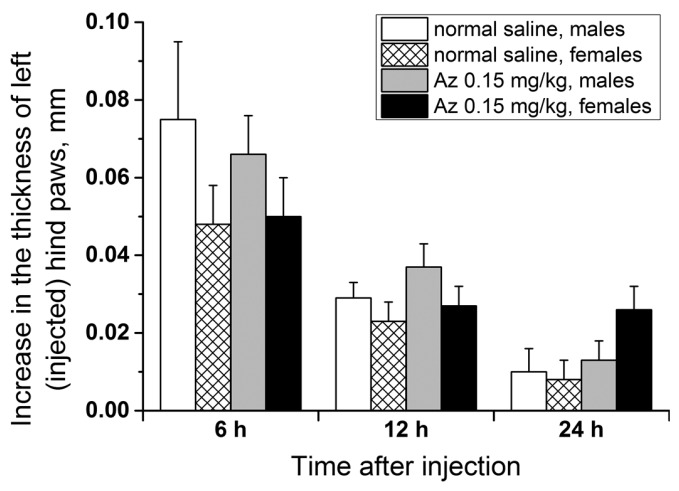
The capacity of Az (0.15 mg/kg subcutaneously) to provoke allergic (a delayed-type hypersensitivity) reaction in male and female ICR mice. The differences in the thickness of the experimental left (injected with Az) and the control right hind paws are presented for animal groups with preliminary Az sensitization and without it 6, 12 and 24 h after the second peptide administration. There was no significant difference in the increase in the thickness of left hind paws between all animal groups (*p* > 0.05, Kruskall-Wallis ANOVA on ranks). The results are presented as mean values ± SEM, *n* = 10.

To test the allergenicity of Az, its ability to induce a delayed-type hypersensitivity reaction in male and female ICR mice at a dose of 0.15 mg/kg was investigated. The scheme of animal immunization (the 1st day subcutaneously and the 5th day in a left hind paw) was similar to the test with trinitrobenzenesulfonic acid but Az was used as an immunogen. In the control groups, the animals were first given the sodium chloride physiological solution (normal saline) subcutaneously and after 5 days were similarly injected with Az in a left hind paw. The degree of left hind paw edema was estimated 6, 12 and 24 h after the second Az injection relatively to the control paw size (Figure 8). In all groups of animals, a small swelling of the experimental paws was observed 6 h after the second Az injection. After 12 h the revealed edema decreased and after 24 h there was practically no swelling. The size of the edema and its dynamics were not significantly different between the animal groups with preliminary Az sensitization and without it. In all tests carried out, no allergic reaction was detected. Thus, Az at a dose of 0.15 mg/kg demonstrated no allergic effect in the delayed-type hypersensitivity reaction test in ICR mice.

### 2.9. Mutagenicity of Az

To study Az mutagenicity in vitro, its ability to induce mutations in the hypoxanthine guanine phosphoribosyltransferase (hprt) gene of mammalian CHO-k1 cells was tested. This is the common assay for detection of gene mutations in mammalian cells [15]. For this purpose, Az was added to the cell growth medium at concentrations ranging from 2.8 to 2000 μg/mL for four hours in the presence or absence of a metabolic activation system (S9 mixture [16]) and after a cultivation period of 8 days, the cells were sub-cultured in the presence of a specific cytostatic agent 6-thioguanine. The inactivation of hprt gene due to any induced mutations led to the resistance of CHO-k1 cells to the cytostatic effect of this purine analog and allowed the selection and counting of the mutant cell colonies grown in its presence.

As positive controls, two high-mutagenic agents were used: ethylnitrosurea (6.25 and 12.5 μg/mL) and methylcholanthrene (2.5 and 5 μg/mL) in the absence and in the presence of the metabolic activation system, respectively. To determine the basic level of spontaneous mutations in the hprt gene, CHO-k1 cells were cultivated in the intact growth medium before their sub-culturing in the presence of 6-thioguanine. The mean frequency of spontaneous mutations was evaluated as 25.2 ± 1.6 × 10^−6^. Although for all positive control conditions the statistically significant rise (*n* = 6, *p* < 0.05, Kruskall-Wallis ANOVA on ranks) in the frequency of mutations in the hprt gene was observed (33.9–39.5 × 10^−6^ for ethylnitrosurea and 35.7–45.7 × 10^−6^ for methylcholanthrene), Az in all tested concentrations (up to 2000 μg/mL) did not provoke any significant increase over a basic level of spontaneous mutations in this gene (23.5–30.2 × 10^−6^, *p* > 0.05). Thus, in this cell system Az did not demonstrate any mutagenic capacity.

## 3. Discussion

As discussed above peptide and protein drugs in many cases possess higher efficacy and better specificity as compared to low molecular weight compounds. The Az, manifesting specific inhibiting activity against muscle nAChR, is a good candidate to be a local muscle relaxant. However, to claim Az as a perspective medicine, it is necessary to check a number of its biological characteristics, including muscle relaxing efficacy, acute and chronic toxicity, pharmacokinetics, mutagenicity, immunotoxicity and allergenicity. The current study was undertaken to determine if Az has a perspective to be used as a local muscle relaxant and it was carried out according to General requirements to conduct preclinical studies of drugs as stated in Appendix No. 7 to the Rules of Good Laboratory Practice of the Eurasian Economic Union in the field of drug circulation [17].

Basing on these requirements, we have studied the pharmacology and in vivo efficacy of the Az, its pharmacokinetics and toxicology, including toxicity after its single and repeated administration, specific toxicity and mutagenicity. Earlier in competition experiments with radioactive α-bungarotoxin, Az showed high affinity to muscle-type *Torpedo* nicotinic acetylcholine receptor (nAChR) (IC_50_ 0.18 ± 0.03 μM) and lower efficiency to human α7 nAChR (IC_50_ 22 ± 2 μM) [1]. In *Xenopus* oocytes heterologously expressing human muscle-type nAChR it was more potent against the adult form (α1β1εδ, IC_50_ 0.44 ± 0.1 μM) than the fetal form (α1β1γδ, IC_50_ 1.56 ± 0.37 μM). In the present study, we have found that Az exhibited high affinity for mouse muscle α1β1εδ nAChR (IC_50_ 19 ± 8 nM) but was less potent to human α7 nAChR (IC_50_ 2.67 ± 0.02 μM) in calcium imaging assay. It was more active than rocuronium against both muscle and α7 nAChR and manifested higher selectivity to muscle receptor (about 140 times) as compared to rocuronium (about 100 more active to muscle type). In general, these data are in agreement with earlier results. At concentrations up to 100 μM, Az had no effect on heteromeric rat α4β2 or human α3-containing nAChRs (α3β2, α3β4, etc.). It also showed no activity against 5-HT_3_ receptors at concentration up to 10 μM and GABA_A_ (α1β3γ2 or α2β3γ2) receptors at concentration up to 100 μM [1]. All these data show high Az selectivity to muscle type nAChR. It should be noted that the nondepolarizing neuromuscular blocking agents now used as muscle relaxants reversibly and concentration-dependently inhibited in the low micromolar range the neuronal nAChRs, including α3β2, α3β4, α4β2 and α7 subtypes [18]. In our experiments, rocuronium dose-dependently inhibited neuronal α3-containing nAChRs. The mechanism (i.e., competitive vs. noncompetitive) of the block at the neuronal nAChRs was dependent both on the receptor subtype and the agent tested. Our data indicate that Az is more selective to muscle type nAChR than currently used relaxant, therefore it may produce less side effects in practice.

To determine the muscle relaxing capacity of Az, its influence on the forelimb grip strength of male ICR mice was studied. Grip strength test is a widely used non-invasive method to quantify objectively the muscular strength of mice and rats and to investigate the effects of neuromuscular disorders and drugs. It is based on the natural tendency of a rodent to grasp a bar or grid when it is suspended by the tail [19,20]. It was found that a single Az injection in mouse forelimb muscles resulted in the decrease of its grip strength. The effect was dose-dependent and the strongest decrease was observed at the highest dose used (0.3 mg/kg). The effect was evident 5 min after administration and maintained for 25–55 min depending on the dose. For comparison, we have tested nondepolarizing muscle relaxant rocuronium and found that it was very poor in grip strength test. Its effect was extremely fast and disappeared with 5 min after injection. Moreover, at a dose of 0.13 mg/kg it was lethal to mice and at 0.08 mg/kg produced no statistically significant relaxing effect as compared to control. Thus, Az demonstrated much better performance in grip strength test. At 0.1 mg/kg (the intermediate dose used) the Az effect was slightly more pronounced as compared to other nondepolarizing muscle relaxant pancuronium, the decrease being by 37% for Az and by 24.1% for pancuronium [20]. However, taking into consideration the 4.4-fold molecular mass difference, Az is much more active than pancuronium; 0.1 mg/kg corresponds 39 and 175 nmoles/kg, respectively. In grip strength test the relaxant activity of botulinum neurotoxins was assessed in rats [20]. In this test, the botulinum neurotoxin was more active and its effect was much more persistent. At 0.24 U of neurotoxin injected intramuscularly, the normal strength was not observed for more than 14 days [21]. This long muscle function disturbance might not be beneficial at some practical applications.

To study pharmacokinetics, the radioiodinated Az analogue was prepared. The only amino acid residue, which may be iodinated in the peptide, is histidine. Oxidative conditions used for iodination may result in oxidation of two tryptophan residues present in Az molecule. Therefore, to obtain the isotope-labeled derivative, a three-step procedure was chosen, including the introduction of formyl protective groups in the tryptophan residues, iodination of the protected Az using the iodide/chloramine T mixture and deprotection of the obtained derivative under alkaline conditions. The radioactive analogue possessing high radioactivity was used for the pharmacokinetics study. No lethality was observed at intravenous injection of [^125^I]-Az at doses of 0.25 and 0.5 mg/kg. While the dose of 0.5 mg/kg is very close to LD_50_ of Az (0.51 mg/kg), it induced no death in injected mice. This fact may be explained by the lower toxicity of iodinated Az. Earlier we have shown that histidine residue is essential for Az activity [1]; its replacement by alanine resulted in strong decrease in capacity to bind to *Torpedo* nicotinic acetylcholine receptor. Thus, introduction of iodine in histidine residue may result in the decrease of [^125^I]-Az toxicity.

Pharmacokinetics and pharmacodynamics are the empirical mathematical models that describe the time course of drug effect after administration [22]. Pharmacokinetics describes the disposition of drug in the organism, while pharmacodynamics relates the drug effects to their concentration in the plasma and at the site of action. They can be used to predict the drug action at different doses and by this way to optimize the safe and effective use of the drug. Information from pharmacokinetic studies can be used in the design and analysis of data from other toxicity studies. Several pharmacokinetics parameters for Az were determined including AUC(0 → t), C_max_ and T_1/2_. It was found that a higher Az concentration was achieved in the blood after its intravenous injection. We were not able to find in the available literature pharmacokinetics parameters obtained for any peripherally acting muscle relaxant in mice. However, there are several studies published for humans [23,24,25,26]. It should be noted that the results obtained on different species with different administered doses and different analysis methods cannot be compared adequately. Nondepolarizing peripherally acting muscle relaxant can be classified as long-acting, intermediate- and short-acting blockers [26]. By its pharmacokinetics parameters, Az is more similar to intermediate-acting relaxants. Its excretion half-life was estimated as 15–40 min depending on administration way, while for intermediate-acting relaxants in human it varied from 17 min (atracurium [27]) to 71 min (rocuronium [28]). For Az, C_max_ was 745 ng/mL at intravenous injection (0.5 mg/kg); this value was about 1 µg/mL for vecuronium [29] and 27 µg/kg for rocuronium [23]. It should be noted that for Az we observed fairly good correlation between the time of elimination (half-life 15–40 min) and duration of muscle relaxant effect (25–55 min).

Acute toxicity of Az was determined using different administration ways: intraperitoneal (ip), intramuscular (im) and intravenous (iv) injections. The highest toxicity was observed at iv injection (LD_50_ 0.51 mg/kg). The LD_50_ of rocuronium at iv injection to rats is about 0.3 mg/kg [30], for (+)-tubocurarine in mice—0.11 mg/kg [31] and for vecuronium in mice—50 µg/kg [32]. In our experiment, intramuscular injection of rocuronium at dose of 0.13 mg/kg resulted in the death of the animal, while Az at 0.7 mg/kg was not lethal. These data indicate that the Az possesses lower acute toxicity than common peripherally acting muscle relaxants. It should be noted that the surviving animals have fully recovered in a fairly short period of time. This is a standard feature of curare-like drugs and can be considered in favor of their application in practice.

The subchronic toxicity of Az was studied at its intramuscular administration in two doses of 0.1 and 0.5 mg/kg for 14 days. Among a number of different parameters investigated, several were found to be influenced by Az administration. Thus, in males receiving Az the differences from control males receiving the carrier were observed in the following parameters: the relative heart mass was increased by 0.076%, the triglyceride level was decreased by 0.28 mmol/L and the platelet count was increased by 81 g/L. In the group of females treated with the drug at a dose of 0.1 mg/kg, the differences from the control animals in some biochemical parameters were also found—the cholesterol level was lowered by 0.73 mmol/L and calcium concentration by 0.11 mmol/L. However, these changes did not exceed the physiological norm and fall within the range of normal physiological values for male and female rats [14]. It would be incorrect to speak in this case about the toxic effects of Az, since the fluctuations of the parameters are within the range of the physiological norm. For the same reason, we cannot say that there are differences in the effects of Az on males and females. No other signs of toxicity were observed during chronic intramuscular administration of Az to female and male rats within a two-week withdrawal period. Therefore, we concluded that peptide had no significant chronic toxicity in rats at doses tested.

Like some other chemical substances, Az may induce undesirable immune reaction or allergy. Immunotoxicity is defined as adverse effects on the functioning of the immune system that result from exposure to chemical substances. The adverse effects on the immune system include reduction in antibody production, reduction in cytokine secretion, hypersensitivity and some other effects [33]. Altered immune function may lead to the increased incidence or severity of infectious diseases or cancer, since the immune system’s ability to respond adequately to invading agents is suppressed. Identifying immunotoxicants is difficult because chemicals can cause a wide variety of complicated effects on immune function. That is why several methods were used in this work to estimate the immunotoxicity of azemiopsin. The precise testing of immunotoxicity and allergenicity is required to estimate the potential hazard of the putative drug. Immunotoxicity assays are important tests for new drugs being developed for application in the humans. Considering the complexity of the immune response, in vivo studies are more relevant. In this work, three different in vivo assays were used to assess Az immunotoxicity (Section 2.8). None of them revealed immunotoxic capacity of Az. No signs of allergenicity was seen in any of the in vivo tests described above; therefore, we decided first to try a single dose of 0.15 mg/kg, which is close to the anticipated therapeutic one. Allergenicity of Az was checked as its ability to induce a delayed-type hypersensitivity reaction. This test did not reveal any allergenicity signs as well, then for ethical reasons we considered additional studies inappropriate. It should be noted that neuromuscular blocking agents contribute to 50–70% of allergic reactions during anesthesia [34]. Suxamethonium appeared to be more frequently involved, while, pancuronium and cis-atracurium are associated with the lowest incidence of anaphylaxis [34]. An increased frequency of allergic reactions to rocuronium was recently noted [35]. As no allergic reaction to Az was observed in our study it may have advantages over other relaxants in this respect.

Mutagenicity, that is the induction of permanent transmissible changes in the amount or structure of the genetic material of cells or organisms, is very important parameter of drug candidate. Highly mutagenic compounds can hardly be considered for drug development, therefore mutagenicity studies are a necessary phase in preclinical evaluations. In vitro Az mutagenicity studies using mammalian CHO-k1 cells showed no mutagenic capacity. While studies of rocuronium using cultured human peripheral blood lymphocytes indicated that it was capable of causing genotoxicity via clastogenic effects at concentrations at which a significant cytotoxic effect does not occur [36]. Thus, Az is safer as compared to rocuronium.

The main application of NMRs is their use in surgery to relax the muscles during operative interventions. They are administered during anesthesia and allow to reduce the dose of anesthetics thus decreasing their adverse effects. There are some general requirements to NMR and ideally, it should have a rapid onset and short duration of action, no cardiovascular side effects, no accumulation in the body, no active metabolites, organ-dependent drug metabolism and elimination as well as an available and adequate antagonist [37,38]. Az satisfies most these requirements: it is fast acting agent with relatively short duration of action, does not accumulates in the body and has no active metabolites. All this allows considering Az for the possible application as NMR.

As it was described in introduction, other area for muscle relaxant application is the treatment of dystonia. Nowadays the main drug for dystonia treatment is extremely toxic botulinum toxin. In addition to high toxicity, there are several other side effects associated with its application. Az at non-lethal doses showed good muscle relaxing activity (Figure 3). It is deprived of some shortcomings (e.g., long action period) inherent in the botulinum toxin and may be regarded as a candidate for dystonia treatment.

The above considerations allow to conclude that Az has good drug-like properties for the application as local muscle relaxant, however further studies should be conducted to confirm it safety and applicability including investigation on humans.

## 4. Conclusions

In summary, we investigated the preclinical profile of Az in regard to in vitro and in vivo efficacy, acute and chronic toxicity, pharmacokinetics, allergenic capacity, immunotoxicity and mutagenic potency. Our in vitro studies confirmed the high affinity and specificity of Az for muscle type nAChR. The peptide effectively inhibited muscle nAChR but was less potent at α7 nAChR and had a low affinity to α4β2 and α3-containing nAChR, as well as to GABAA or 5HT3 receptors. Its muscle relaxant effect was demonstrated at intramuscular injection to mice at doses of 30–300 µg/kg, the relaxant activity being higher than that of commonly used peripheral muscle relaxants. The highest acute toxicity was observed at intravenous injection of Az, at intramuscular or intraperitoneal administration it was less toxic. No toxicity signs were observed at doses inducing muscle relaxant effects. Toxicology studies in mice indicated that Az was well tolerated during chronic dosing and showed no immunotoxicity, allergenic or mutagenic activity, which differ if from most currently used muscle relaxants. Overall, the results demonstrate that azemiopsin has good drug-like properties for application as a local muscle relaxant and in its parameters is not inferior to the relaxants currently used. It should be noted that Az possessed narrow therapeutic window, which is a typical characteristic and a weak point for all muscle relaxants with a similar mechanism of action. Modifications are required that will not affect (or preferably increase) the effectiveness of the drug but reduce its toxicity and extend its narrow therapeutic window.

## 5. Materials and Methods

### 5.1. Materials

A polystyrene-poly(ethylene glycol) 2000 block-copolymer resin, modified with Knorr linker Tentagel S RAM was from Rapp Polymere GmbH (Tübingen, Germany). Fmoc-protected amino acids, 4-methyl piperidine were from Mosinter (Ningbo, China). 1-[Bis(dimethylamino)methylene]-1H-1,2,3-triazolo[4,5-b]pyridinium 3-oxid hexafluorophosphate (HATU), 1-Hydroxy-7-azabenzotriazole (HOAt) and DL-Dithiothreitol (DTT) were from DEMO Medical (Shanghai, China). *N*,*N*-diisopropylethylamine (DIPEA) was from Iris Biotech GmbH (Marktredwitz, Germany), trifluoroacetic acid from Solvay S.A. (Bruxelles, Belgium), normal saline (sterile 0.9% NaCl solution), complete Freund’s adjuvant and trinitrobenzenesulfonic acid (TNBS) from Sigma-Aldrich Chemie Gmbh (Munich, Germany), bovine serum albumin (BSA) from Amresko, Rocuronium Bromide from Fresenius Kabi (Bad Homburg, Germany). All other reagents and solvents of the highest purity available were purchased from local manufacturers and used without additional purification.

### 5.2. Animals

Specific pathogen-free (SPF) ICR mice (6–8 weeks old, weight 29–34 g) and SD rats (9–11 weeks old, weight of males 254–310 g, weight of females 188–220 g) of both sexes were obtained from the Animal House of the Branch of the Shemyakin-Ovchinnikov Institute of Bioorganic Chemistry, Russian Academy of Sciences and used for studies in vivo. Animals were housed in groups of 5–6 mice or 2 rats at 20–25 °C, 30–70% relative humidity and under a 12-h light-dark cycle (lights on at 08:00). Standard chow for rodents and filtered tap water were provided *ad libitum*. All studies involving animals were approved by the Institutional Animal Care and Use Committee (IACUC) of the Branch of the Shemyakin-Ovchinnikov Institute of Bioorganic Chemistry, Russian Academy of Sciences, the experimental protocol codes are No. 528/16, 531/16, 534/16, 560/16, 576/17, 577/17. The dates of approval are 31 March 2016, 20 April 2016, 20 May 2016, 15 September 2016, 10 February 2017, 10 February 2017, respectively. All solutions for in vivo studies were prepared fresh before the administration: the necessary amount of freeze-dried Az was dissolved in normal saline (0.9% sterile NaCl solution).

### 5.3. Az Synthesis

#### 5.3.1. Solid Phase Az Synthesis

Az synthesis was performed on automatic peptide synthesizer based on Gilson automated liquid handler system according to Gilson application note 228. The peptide was synthesized utilizing a solid phase methodology with Fmoc/t-Bu protection scheme. A polystyrene-poly(ethylene glycol) 2000 block-copolymer resin Tentagel S (extent of loading 0.3 meq/g) was modified with Knorr linker followed by peptide chain assembly. A 4-fold excess of protected amino acids was used; condensation reagent was HATU/HOAt in amount equimolar to that of protected amino acids and activation reagent—2.4 equivalents of DIPEA. Coupling time for 7 C-terminal amino acid residues (up to Pro15) was 1 h and for subsequent residues the coupling time was increased to 2 h. For His9 and Pro15 residues a repetitive coupling was required to achieve their complete acylation. After chain assembly, peptidyl-polymer was subjected to a total deprotection/cleavage by the treatment with 12 mL of reagent L [39] per 1 g of dry resin for 2 h. After that, the resin was filtered, washed with trifluoroacetic acid and combined filtrate was evaporated under vacuum to ca. 30% of initial volume. The residue was diluted tenfold with dry diethyl ether; the precipitated crude peptide was filtered out, washed with ether and dried under vacuum. The crude peptide was dissolved in starting buffer and 1 g was applied on a ECOPlus column (35 × 250 mm) packed with 60 mL of SPS-Bio CM (12 μm, Purolite Corporation, Bala Cynwyd, PA, USA) resin. The column was eluted with 8 column volumes of linear concentration gradient of ammonium bicarbonate from 50 mM to 1 M (pH 8.9) in 10% isopropyl alcohol. Fractions containing peptide were collected and isopropyl alcohol was evaporated under vacuum, the remaining solution was freeze-dried. Freeze-dried peptide was dissolved in water to a final concentration of 50 mg/mL, titrated with acetic acid to a pH 3.5 and applied on a Thermo Scientific Hypersil GOLD aQ (12 µm, 250 × 50 mm) column. Elution was carried out with a linear gradient of acetonitrile in water from 10 to 35% in 30 min in the presence of 1% acetic acid at a flow rate of 150 mL/min. Main fraction was collected and freeze-dried; the obtained azemiopsin acetate had a purity greater than 97% as confirmed by UPLC-MS analysis. The total yield of pure peptide was about 20%, based on resin loading.

#### 5.3.2. Formylation of Az

For this modification, the peptide was dissolved in formic acid at a concentration of 50 mg/mL, to the resulting solution hydrogen chloride in formic acid was added to a final concentration of 200 mM HCl. The mixture was stirred for 12 h, then formic acid was evaporated on a rotary evaporator, the residue was dissolved in water and freeze-dried. The formylation was complete, as evidenced by HPLC-MS analysis.

#### 5.3.3. Iodination of Formylated Az

The iodination was carried out in general according to the published procedure [13]. In brief, 500 μL of diformyl azemiopsin solution in water (2.2 mM) were mixed with 100 μL of 1 M Tris-HCl buffer (pH 6.8), 30 μL of Na^127^I solution (100 mM) and 15 μL of Na^125^I solution with specific radioactivity of 2000 Ci/mmole. Then 27 μL of a 100 mM chloramine T solution was added to the mixture, the solution was mixed vigorously and incubated for 30 min at room temperature. The radioiodinated product was isolated by HPLC on Jupiter C18 column (10 µm, 10 × 250 mm, Phenomenex) in a linear gradient of acetonitrile in water from 10 to 25% in 15 min in the presence of 0.1% trifluoroacetic acid at a flow rate of 1 mL/min. The fraction containing diiodinated diformyl azemiopsin was concentrated on Savant SpeedVac Centrifuge Concentrator SVC100D and the solution obtained was used for deprotection.

#### 5.3.4. Deprotection of Radioiodinated Az

To remove formyl protecting groups, the radioiodinated Az derivative was treated with 100 mM sodium hydroxide solution for 12 h. HPLC-MS analysis showed almost complete (>90%) removal of the formyl groups. After deblocking, the reaction mixture was neutralized with dilute hydrochloric acid, giving a solution of diiodoazemiopsin in an isotonic solution of sodium chloride. The concentration of Az in the resulting solution was determined spectrophotometrically from the absorbance at 280 nm and the radioactivity was measured using Wizard 1470 Automatic Gamma Counter (Perkin Elmer, Waltham, MA, USA). The specific radioactivity of the derivative was 0.15 Ci/mmole.

### 5.4. Toxicity Studies

#### 5.4.1. Acute Toxicity

##### Az Acute Toxicity

Az acute toxicity was estimated for its intravenous (iv) and intramuscular (im) administration to male ICR mice in a stepwise procedure.

For the iv route of administration, 20 mice were randomly divided in 5 groups of 4 animals each. The animals of each group received a single dose of Az (0.3, 0.4, 0.5, 0.6, or 0.7 mg/kg, respectively) injected into the lateral tail vein at a volume of 1 mL/kg. For the im route of administration, first two experimental groups of 3 animals each were formed. Az at doses of 0.75 and 1.0 mg/kg, respectively, was injected into the quadriceps muscle of the thigh (quadriceps femoris muscle) of two mouse hind limbs at a volume of 0.5 mL/kg to each muscle. Then, five experimental groups of 5 animals each were formed. Az was injected into the quadriceps muscle of the thigh (quadriceps femoris muscle) of the two hind limbs of male ICR mice at doses of 0.8, 0.775, 0.75, 0.725 and 0.7 mg/kg. The injection volume to each limb was 1 mL/kg. Further, neurotoxic manifestations were recorded in animals at 5, 10, 20, 30, 60 and 90 min and 24 h after administration using the functional observation battery, the number of death was counted as well.

After iv and im injections of Az, the mice were observed for 24 h and signs of toxicity or lethality were recorded. For the iv and im route of administration, the median lethal dose (LD_50_) was calculated using a probit analysis [40]. For the im route of administration, the maximum tolerated dose was determined.

##### Rocuronium Acute Toxicity

Rocuronium was administered intramuscularly at doses of 0.13 mg/kg (*n* = 1), 0.1 mg/kg (*n* = 4) and 0.08 mg/kg (*n* = 4) in the triceps of the forelimbs of male ICR mice (8–9 weeks old). Control animals were injected with saline (*n* = 4). The volume of administration was 0.5 mL/kg in each limb.

#### 5.4.2. Subchronic Toxicity

A repeated dose 14-day intramuscular toxicity study was conducted on 36 SD male and 36 SD female adult rats. They were divided into 3 groups. The first control group was given normal saline, the second and the third experimental groups received Az at doses of 0.1 mg/kg and 0.5 mg/kg, respectively, daily for 14 days. The substances were injected into the quadriceps muscle of the thigh. Every day the animals were examined and any clinical signs of intoxication, body weight and food intake were recorded. The one half of the animals were euthanized on the 15th day of the study, the second half—after a 2-week cancellation period on the 29th day of the study. All animals were autopsied and their organs were inspected for any pathological signs, weighed and histologically examined. The ratio of organ-to-body weight was calculated for several organs: brain, heart, liver, spleen, thymus, kidneys, lungs, testicles and ovaries. The histological analysis was carried out for a number of organs: kidney, adrenal glands, testis, ovary, spleen, thymus, brain, heart, lung, liver, lymph node (mesenteric and mandibular), stomach, skin and muscle from the site of the administration.

A biochemical analysis of blood serum parameters was performed. A level of aspartate aminotransferase, alanine aminotransferase, glutamate dehydrogenase, alkaline phosphatase, gamma-glutamyl transferase, urea nitrogen, creatinine, total bilirubin, total protein, albumin, globulin, phosphorus, calcium, total cholesterol, triglycerides, albumin/globulin ratio was estimated. The analysis was performed using Randox GB reagent kits for each tested parameter and the automatic biochemical analyzer Sapphire-400 (Tokyo Boeki Ltd., Toyko, Japan).

A hematological analysis of animal blood included the evaluation of red and white blood cell number, a hemoglobin level, hematocrit, red cell distribution width, mean corpuscular volume (MCV), mean corpuscular hemoglobin (MCH), mean corpuscular hemoglobin concentration (MCHC), platelets number, a mean platelet volume, a mean platelet component and a cell number of neutrophils, eosinophils, basophils, lymphocytes, monocytes, large unstained cells, reticulocytes. The analysis was performed using a hematological analyzer Mythic 18 Vet (C2 DIAGNOSTICS S.A., Montpellier, France).

### 5.5. Pharmacokinetics

The pharmacokinetic study was performed using 163 male ICR mice. Four experimental groups with 40 animals in each were formed. Three intact mice were used as control animals. The animals of the first two experimental groups were intravenously injected with 0.25 and 0.50 mg/kg [^125^I]-Az, respectively. The animals of the other two experimental groups were intramuscularly injected with 0.25 and 0.50 mg/kg [^125^I]-Az, respectively. To estimate Az elimination rate, animal blood samples were taken from the orbital sinus 5, 15, 30, 60 min and 1, 2, 4 and 24 h after [^125^I]-Az administration. The obtained blood samples were weighed. For each indicated time point, [^125^I]-Az concentration was calculated as a mean value in blood samples of five animals. The radioactivity (cpm) in the blood samples was counted using a Wallac 1470 WIZARD^®^ Gamma Counter (Perkin Elmer, Waltham, MA, USA). The specific radioactivity of [^125^I]-Az was 6.26 × 10^7^ cpm/mg. Radioactivity data (cpm) were re-calculated to the concentration of [^125^I]-Az in the obtained blood samples. The specific pharmacokinetic parameters (AUC (0 → t), C_max_, T_1/2_) of [^125^I]-Az were estimated.

### 5.6. Mutagenicity

The ability of Az to induce mutations in the hypoxanthine guanine phosphoribosyltransferase (hprt) gene of Chinese hamster ovary CHO-k1 cells was tested. The cells were purchased from the Russian collection of cell cultures (Institute of Cytology, Russian Academy of Sciences, Saint Petersburg, Russia). CHO-k1 cells were cultured in the growth medium DMEM/F12 with high glucose, glutamine and without Na_2_CO_3_, HEPES (Sigma-Aldrich Chemie Gmbh, Munich, Germany) supplemented with 10% FBS (BioSera, Nuaille, France), 0.1 M HEPES, 80 mg/mL gentamicin and 10 mg/mL fluconazole at 37 °C, 5% CO_2_ in a CO_2_ incubator. Before Az treatment CHO-k1 cells were subcultured and incubated in HAT medium (5 mM hypoxanthine, 20 mM aminopterin and 0.8 mM thymidine (Sigma-Aldrich Chemie Gmbh, Munich, Germany)) for three days, then the medium was changed and they were cultured for one day in HT medium (5 mM hypoxanthine and 0.8 mM thymidine (Sigma-Aldrich Chemie Gmbh, Munich, Germany)). After that, the cells were subcultured at a density of 40,000 cells per cm^2^ in 10-cm Petri dishes. Next day they were treated for 4 h with different Az concentrations ranging from 2.8 to 2000 μg/mL in the presence or absence of a metabolic activation system (S9 mixture [15]). As positive controls, two high-mutagenic agents were used: ethylnitrosurea (6.25 and 12.5 μg/mL) and methylcholanthrene (2.5 and 5 μg/mL) in the absence and in the presence of the metabolic activation system, respectively.

After the treatment, the CHO-k1 cells were partly subcultured at a density of 150–500 cells per 55 cm^2^ to determine the cytotoxicity of the different Az doses and control substances. The substance cytotoxicity was determined by a relative survival (RS) capacity of the cells, calculated as a ratio between the cloning efficiency (CE) of the cells plated immediately after the treatment and the normal cellular CE of non-treated cells (negative controls) after 7 days of culturing.

$$RS=\frac{CE\;of\;the\;treated\;cells}{CE\;of\;non-treated\;cells}\times 100$$

$$CE=\frac{Number\;of\;colonies}{Number\;of\;cells\;plated}$$

The rest of the treated CHO-k1 cells were maintained in the growth medium for 8 days to allow near-optimal phenotypic expression of any induced in hprt gene mutations. Then, to determine the frequency of the induced mutations, the cells were subcultured and maintained in the growth medium in the presence (2,000,000 cells) or in the absence (500 cells) of a selective agent (2.2 µg/mL 6-thioguanine) for 7 days. After that, the number of cell colonies in both media was counted and the frequency of mutations was calculated with the formula:

$$Mutation\;frequency=\frac{CE\;of\;mutant\;cells\;in\;a\;selective\;medium}{CE\;of\;cells\;in\;a\;non-selective\;medium}$$

### 5.7. Immunotoxicity

Three separate immunotoxicity tests were performed: (1) evaluation of cellular immunity in a delayed-type hypersensitivity test, (2) evaluation of animal immune response to a standard antigen, (3) evaluation of the phagocytic activity of peritoneal macrophages.

For carrying out these experiments 90 male ICR mice were used, they were divided equally into three groups (30 animals per group) for each study and in these groups they were subdivided into subgroups of 10 mice each and treated as follows: mice of the first subgroup were injected with normal saline im, the second subgroup animals were treated with Az (0.15 mg/kg im) and the animals of the third subgroup were treated with Az (0.50 mg/kg im). The drugs were injected into the quadriceps muscle of the thigh (musculus quadriceps femoris) daily for seven days (1 mL/kg).

#### 5.7.1. Evaluation of Cellular Immunity in a Delayed-Type Hypersensitivity Test

On the last 7th day of the Az administration, the mice of the first group (30 animals) were immunized with a solution of trinitrobenzenesulfonic acid (TNBS) (200 μL, 10 mM) subcutaneously at the base of a tail*.* After 6 days the animals were secondarily immunized with TNBS (50 μL, 10 mM) injected in the pad of the left hind paw, the same volume of normal saline was injected into the right hind paw. 24 h after the second immunization, the animals were euthanized (CO_2_ inhalation) and the weights of their experimental and control paws were determined. The reaction index was calculated using the indicated formula:

$$R_{i}=\frac{W_{exp}-W_{cont}}{W_{exp}}\times 100\%$$

*R_i_*—reaction index, *W_exp_*—weight of the experimental paw, *W_cont_*—weight of the control paw.

#### 5.7.2. Evaluation of Animal Immune Response to a Standard Antigen

On the last 7th day of the Az administration, the mice of the second group (30 animals) were immunized intraperitoneally with a 1:1 mixture of 200 μL of bovine serum albumin (BSA, 0.5 mg/mL) with a complete Freund’s adjuvant. 10 days after the 1st immunization, the mice received the second 200 μL ip injection of the antigen (BSA, 0.5 mg/mL) in an incomplete Freund’s adjuvant (1:1 ratio). After 7 days the venous mouse blood was collected from the inferior vena cava, then the blood serum was isolated and the titers of IgG antibodies to BSA were determined using a standard enzyme immunoassay.

#### 5.7.3. Phagocytic Activity of Peritoneal Macrophages

On the next day after the seven-day course of the Az administration, the mice of the third group (30 animals) were injected intraperitoneally with 2 mL of ink particles suspension. After 10 min the mice were euthanized (CO_2_ inhalation) with a subsequent isolation of the peritoneal exudate from their abdominal cavity. In the exudate (1 μL) the total number of peritoneal macrophages and the number of the ink particle-containing (phagocytic) cells among them were counted using a Gorjaev’s chamber.

### 5.8. Allergenicity

Allergenicity of Az was studied in a delayed-type hypersensitivity test on male (20 animals) and female (20 animals) adult ICR mice. The mice were divided into four groups (two control and two experimental groups) of 10 animals (males or females) per group. Mice of the experimental groups were injected subcutaneously at the base of a tail with Az solution (0.15 mg/kg) emulsified in Freund’s complete adjuvant in a ratio of 1:1 (2 mL/kg). Similarly, the mice in the control groups were administered with a suspension of normal saline in Freund’s complete adjuvant. Five days later all animals received an injection of Az (0.15 mg/kg, 1.33 mL/kg) in the pad of the left hind paw. To estimate the intensity of the studied allergic reaction, 6, 12 and 24 h after the second Az injection the thickness of the left and right hind mouse paws was measured with a digital caliper.

### 5.9. Efficacy and Specificity Studies

#### 5.9.1. Neuroblastoma Cell Culturing and Transient Transfection

Human neuroblastoma cells SH-SY5Y were cultured in DMEM/F12 medium (ThermoFisher Scientific, Waltham, MA, USA) supplemented with 10% fetal bovine serum (FBS) (PAA Laboratories GmbH, Pasching, Austria), 2.5 μg/mL amphotericin B and 50 μg/mL gentamicin in a CO_2_ incubator at 37 °C and 5% CO_2_ atmosphere. Cells were sub-cultured and plated at a density of 5000–10,000 cells per well in a 96-well black plate (Corning Inc., Corning, NY, USA). They were grown in a CO_2_ incubator for 48–72 h before testing the functional activity of natively expressed nAChRs by calcium imaging.

Mouse neuroblastoma Neuro2a cells were purchased from the Russian collection of cell cultures (Institute of Cytology, Russian Academy of Sciences, Saint Petersburg, Russia). Cells were cultured in DMEM (Paneco, Moscow, Russia) supplemented with 10% FBS. They were sub-cultured the day before transfection and were plated at a density of 10,000 cells per well in a 96-well black plate. On the next day Neuro2a cells were transiently transfected with plasmids coding mouse muscle α1β1δε nAChR (pRBG4-vector) and a fluorescent calcium sensor Case12 (pCase12-cyto vector, Evrogen, Moscow, Russia) in a molar ratio of 2:1 following a lipofectamine transfection protocol (ThermoFisher Scientific, Waltham, MA, USA). Human α7 nAChR (α7 nAChR-pCEP4) was expressed accordingly with a co-expression of the human chaperone Ric-3 (Ric3-pCMV6-XL5, OriGene, Rockville, MD, USA) in a molar ratio 4:1. The transfected cells were grown at 37 °C in a CO_2_ incubator for 48–72 h, before performing the calcium imaging assay.

#### 5.9.2. Calcium Imaging

Calcium imaging procedure was performed as published earlier [11]. Briefly, after removing the growth medium, the transfected Neuro2a cells and cultivated SH-SY5Y cells were washed with a buffer containing 140 mM NaCl, 2 mM CaCl_2_, 2.8 mM KCl, 4 mM MgCl_2_, 20 mM HEPES, 10 mM glucose; pH 7.4. Neuro2a cells expressing muscle nAChR and the protein calcium sensor Case12 were proceeded directly, while SH-SY5Y cells expressing human α3-containing nAChRs natively were loaded with a fluorescent dye Fluo-4, AM (1.824 μM, ThermoFisher Scientific, Waltham, MA, USA) and a water-soluble probenecid (1.25 mM, ThermoFisher Scientific, Waltham, MA, USA) for 30 min at 37 °C and then were kept for 30 min at room temperature according to the manufacturer’s protocol.

To detect the human α7 nAChR-mediated calcium response, transfected Neuro2a cells were incubated with its positive allosteric modulator PNU120596 (10 μM, Tocris Bioscience, Bristol, UK) for 20 min at room temperature before acetylcholine (Sigma-Aldrich Chemie Gmbh, Munich, Germany) addition. To assess mouse muscle or human α3-containing nAChRs this step was skipped. Transfected Neuro2a cells expressing muscle or α7 nAChRs and SH-SY5Y cells expressing α3-containing nAChRs natively were preincubated with Az for 15 minutes at room temperature before agonist addition.

The plates were transferred to the multimodal microplate reader Hidex Sence (Hidex, Turku, Finland) where the cells were excited by light of 485 nm wavelength and emitted fluorescence was detected at 535 ± 10 nm. Fluorescence was recorded every 2 s for three minutes following agonist addition. Responses were measured as peak intensity minus basal fluorescence level and were expressed as a percentage of the maximal response obtained to agonist. Data files were analyzed using Hidex Sence software (Hidex, Turku, Finland) and OriginPro 7.5 software (OriginLab, Northampton, MA, USA, for statistical analysis).

#### 5.9.3. Electrophysiological Experiments

Ovary tissue from adult female *Xenopus laevis* was cut into small pieces and these pieces were digested with collagenase A (4 mg mL^−1^, Worthington Biochemical Corp., Lakewood, NJ, USA) in Barth’s solution without calcium (88.0 mM NaCl, 1.1 mM KCl, 2.4 mM NaHCO_3_, 0.8 mM MgSO_4_, 15.0 mM HEPES/NaOH, pH 7.6) for 1.5 ± 2 h at 20 °C. The oocytes were stored in Barth’s solution with calcium (88.0 mM NaCl, 1.1 mM KCl, 2.4 mM NaHCO_3_, 0.3 mM Ca (NO_3_)_2_, 0.4 mM CaCl_2_, 0.8 mM MgSO_4_, 15.0 mM HEPES/NaOH, pH 7.6) supplemented with 63.0 μg/mL penicillin-G sodium salt, 40.0 μg/mL streptomycin sulfate and 40.0 μg/mL gentamicin. Stage V ± VI oocytes were selected and injected with 3 ng plasmids coding the rat α4 and β2 nAChR subunits (pcDNA3.1 vector) in a molar ratio of 1:1 using an Auto-Nanoliter Injector NanoJect-2 (Drummond Scientific Company, Broomall, PA, USA) in a total injection volume of 23 nL. After injection, oocytes were incubated at 18 °C in Barth’s solution with calcium for 48–120 h. Electrophysiological recordings were made using a Turbo TEC-03X amplifier (npi electronic GmbH, Tamm, Germany) and WinWCP recording software (University of Strathclyde, Glasgow, UK). Oocytes were placed in a small recording chamber with a working volume of 50 μL and 100 μL of ligands (50 μM Az, 20 μM nicotine) solution in Barth’s buffer were applied to an oocyte. Az was pre-applied to an oocyte for 5 min before its co-application with agonist nicotine. To allow receptor recovery from desensitization, the oocytes were superfused for 5–10 min with buffer (1 mL/min) between ligand applications. Electrophysiological recordings were performed at a holding potential of −60 mV.

#### 5.9.4. In Vivo Muscle Relaxant Effect

##### In Vivo Muscle Relaxant Effect of Az

40 male ICR mice were divided equally into four groups: one control and three experimental groups. Animals of the control and experimental groups were treated with normal saline and with Az at doses of 0.03, 0.1 and 0.3 mg/kg, respectively. In addition 6 mice were treated with Az at dose of 0.01 mg/kg. The corresponding solutions were injected into the triceps muscles of the mouse forelimbs at a volume of 0.5 mL/kg per each limb. For all animals their basic forelimb grip strength was recorded before the substance administration with a 1027 grip strength meter (Columbus Instruments, Columbus, OH, USA). Further, their grip strength was measured 5, 10, 15, 20, 30, 60, 90 min after the Az (or normal saline) im administration.

##### In Vivo Muscle Relaxant Effect of Rocuronium

Rocuronium was administered intramuscularly as described for the measurement of acute toxicity (Section 5.4.1). Muscle strength was assessed prior to administration of the substance and then 1, 2, 3, 4, 5 min after administration.

### 5.10. Statistical Analysis

The statistical analysis of the obtained results was performed using Statisticа 7.1 (TIBCO Software Inc., Palo Alto, CA, USA) and OriginPro 9.1 (Microcal, Northampton, MA, USA) software. The data of Az efficacy in vivo were analyzed using one-way repeated measures ANOVA test. To statistically evaluate the efficacy and specificity of Az in vitro, two-tailed Mann–Whitney U test was used. The data of Az subchronic toxicity, immunotoxicity and allergenicity were analyzed using Kruskall-Wallis ANOVA on ranks test. Results are expressed as mean of data ± SEM unless otherwise stated. In all tests, *p* < 0.05 was taken as significant.

## Figures and Tables

**Table 1:** The main pharmacokinetic parameters estimated after a single intramuscular (im) or intravenous (iv) administration of Az to male ICR mice: the area under a pharmacokinetic curve (AUC(0 → t)), the maximum Az concentration in mouse blood (C_max_) and its excretion half-life T_1/2_.

Route/Dose	AUC(0 → t), h × ng/mL	C_max_, ng/mL	T_1/2_, h
im/0.25 mg/kg	328	278	0.30
im/0.50 mg/kg	622	257	0.68
iv/0.25 mg/kg	214	517	0.26
iv/0.50 mg/kg	542	745	0.29

## Supplementary Materials

The following are available online at www.mdpi.com/2072-6651/10/1/34/s1, Figure S1: Muscle relaxant effect of Az at dose of 0.01 mg/kg.
