# Supplementary material for: Azemiopsin, a Selective Peptide Antagonist of Muscle Nicotinic Acetylcholine Receptor: Preclinical Evaluation as a Local Muscle Relaxant
Source: Toxins (Basel). 2018 Jan 7;10(1):34. doi: 10.3390/toxins10010034 (PMC5793121; doi:10.3390/toxins10010034)
Supplement: Supplementary file 1 [file toxins-10-00034-s001.pdf]

Supplementary Materials: Azemiopsin, a Selective Peptide Antagonist of Muscle Nicotinic Acetylcholine Receptor: Preclinical Evaluation as a Local Muscle Relaxant

Irina V. Shelukhina, Maxim N. Zhmak, Alexander V. Lobanov, Igor A. Ivanov, Alexandra I. Garifulina, Irina N. Kravchenko, Ekaterina A. Rasskazova, Margarita A. Salmova, Elena A. Tukhovskaya, Vladimir A. Rykov, Gulsara A. Slasheva, Natalya S. Egorova, Inessa S. Muzyka, Victor I. Tsetlin and Yuri N. Utkin

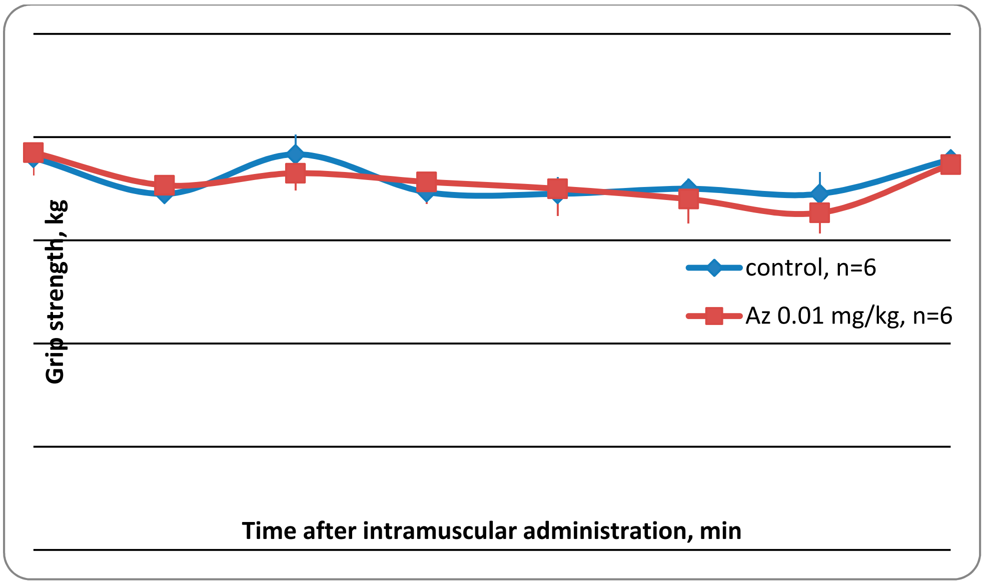

Figure S1. Muscle relaxant effect of Az at dose of 0.01 mg/kg. The time courses of a grip strength of mouse (ICR males) forelimbs at 0–90 min after Az or normal saline (control) intramuscular administration. The results are presented as mean values ± SEM, $n = 6$. No significant differences in the forelimb strength were revealed between control and experimental groups (one-way repeated measures ANOVA $p < 0.05$).
